# Unhealthful Houses

**Published:** 1869-02

**Authors:** 


### UNHEALTHFUL HOUSES.

This is the time of year when New Yorkers are looking out for residences for the coming season, and some of them for a greater permanency. If a family moves into a house, and experiences unusual sickness as to several of its members, it is presumptive evidence that it arises from circumstances connected with the house. Sometimes only certain rooms are pestiferous, from being over a marshy spot, or from being partly under ground, or so attached to another dwelling as to have one side of it almost always damp; at other times the papering of a room has been found a cause of illness. From Jackson to Buchanan, dangerous illness or death has come to every occupant of the Presidential mansion, Mr. Van Buren excepted. Some persons will call it a coincidence, but it is not. It was predicted by medical men in Washington, as a demonstrable result. A bridge was built in Jackson's time, violently opposed by some; the effect was to cause a damming up of water, and to produce a large amount of shallow water and marsh exposure to the sun, the universal cause of chills and fever, and fever and ague, from which Mrs. Pierce suffered greatly, and had to leave her native country, finally dying.

Persons selecting farms in the West, and those having lands and desiring to build dwellings on them, should act with deliberation, and avoid building on northern exposures.

Houses should not be built on spots where fogs are most prone to gather and settle. Sometimes a fog loves the valley, and sometimes the top of a hill; thus some who live in valleys are healthy, and those who live on the hill tops are sickly, and the reverse, and common people are perplexed. It is not the hill tops or the valleys which are healthful in themselves; but where fog, exhalations, malaria, miasm incline to settle, and this is determined by natural laws, there will be disease; let every house site be well drained.

